# Increased Intestinal Permeability Correlates with Sigmoid Mucosa alpha-Synuclein Staining and Endotoxin Exposure Markers in Early Parkinson's Disease

**DOI:** 10.1371/journal.pone.0028032

**Published:** 2011-12-01

**Authors:** Christopher B. Forsyth, Kathleen M. Shannon, Jeffrey H. Kordower, Robin M. Voigt, Maliha Shaikh, Jean A. Jaglin, Jacob D. Estes, Hemraj B. Dodiya, Ali Keshavarzian

**Affiliations:** 1 Department of Internal Medicine, Section of Gastroenterology, Rush University Medical Center, Chicago, Illinois, United States of America; 2 Department of Neurological Sciences, Rush University Medical Center, Chicago, Illinois, United States of America; 3 Center for Brain Repair, Rush Medical College, Chicago, Illinois, United States of America; 4 AIDS and Cancer Virus Program, SAIC-Frederick, Inc., National Cancer Institute-Frederick, Frederick, Maryland, United States of America; University Hospital La Paz, Spain

## Abstract

Parkinson's disease (PD) is the second most common neurodegenerative disorder of aging. The pathological hallmark of PD is neuronal inclusions termed Lewy bodies whose main component is alpha-synuclein protein. The finding of these Lewy bodies in the intestinal enteric nerves led to the hypothesis that the intestine might be an early site of PD disease in response to an environmental toxin or pathogen. One potential mechanism for environmental toxin(s) and proinflammatory luminal products to gain access to mucosal neuronal tissue and promote oxidative stress is compromised intestinal barrier integrity. However, the role of intestinal permeability in PD has never been tested. We hypothesized that PD subjects might exhibit increased intestinal permeability to proinflammatory bacterial products in the intestine. To test our hypothesis we evaluated intestinal permeability in subjects newly diagnosed with PD and compared their values to healthy subjects. In addition, we obtained intestinal biopsies from both groups and used immunohistochemistry to assess bacterial translocation, nitrotyrosine (oxidative stress), and alpha-synuclein. We also evaluated serum markers of endotoxin exposure including LPS binding protein (LBP). Our data show that our PD subjects exhibit significantly greater intestinal permeability (gut leakiness) than controls. In addition, this intestinal hyperpermeability significantly correlated with increased intestinal mucosa staining for *E. coli* bacteria, nitrotyrosine, and alpha-synuclein as well as serum LBP levels in PD subjects. These data represent not only the first demonstration of abnormal intestinal permeability in PD subjects but also the first correlation of increased intestinal permeability in PD with intestinal alpha–synuclein (the hallmark of PD), as well as staining for gram negative bacteria and tissue oxidative stress. Our study may thus shed new light on PD pathogenesis as well as provide a new method for earlier diagnosis of PD and suggests potential therapeutic targets in PD subjects.

**Trial Registration:**

Clinicaltrials.gov NCT01155492

## Introduction

Parkinson's disease (PD) is the second most common neurodegenerative disorder of aging, and is projected to affect nearly 10 million citizens of the world's most populous countries by 2030 [1], [2]. The burden of disability from PD is considerable [3]. Unfortunately there is no optimal treatment for PD and this is at least partly because the majority of patients with PD will be diagnosed and receive treatment after the onset of neurological symptoms when substantial neuronal dysfunction and neuronal loss has already occurred. Thus, a more successful approach could be to diagnose and start treatment before neuronal degeneration results in the emergence of clinical signs of PD. In fact, although the etiology of PD is not known, the pathobiology of neuronal loss in PD is well characterized. It is now well established that the pathological hallmark of PD are neuronal inclusions termed Lewy bodies (LB) or Lewy neurites (LN) whose main component is aggregated and phosphorylated α-synuclein [4], [5]. It is believed that these α-synuclein aggregates are the first steps resulting in neuronal loss that is responsible for neurological symptoms and signs of PD [5]. A better understanding of how α-synuclein aggregates form will be a key for advancing our understanding of the pathogenesis of PD that could lead to early diagnosis and treatment with potentially much better outcome.

While phosphorylated α-synuclein aggregates may be formed as a consequence of oxidative injury [4], the source of neuronal oxidative stress in PD is not known. It is believed that PD pathology is a consequence of interaction between genetic susceptibility and toxic environmental factors [6]. It is highly plausible that the gastrointestinal (GI) tract is a major site and source of oxidative stress in neuronal tissue based on the following: (1) The GI tract is the largest interface between neural tissue and the environment. (2) The GI tract has a large number of neuronal cells in the submucosal plexus and myentric plexus, large enough that the GI neuronal network is called the “second brain” [7]. More importantly, this neuronal network is in close proximity to the potentially injurious factors such as bacterial products capable of inducing oxidative stress [8]. (3) The GI lumen harbors the largest and most diversified human associated microbiota community with the capability of inducing inflammatory and oxidative pathways [9]. The composition of this bacterial community is influenced by both genetic and environmental factors like diet [10]. (4) The GI system and the brain are directly linked anatomically with the dorsal motor nucleus of the vagus nerve, a brain region proposed to express Lewy pathology very early in the disease process [5]; and (5) critically, one important function of the GI tract is to act as a semipermeable barrier, which allows regulation of nutrient, ion and water absorption, and regulates host contact with a large number of dietary antigens and bacterial products [11]. Intestinal permeability can be defined as the facility with which the intestinal epithelium allows molecules to pass through by non-mediated passive diffusion. Several chronic autoimmune intestinal diseases including inflammatory bowel disease and celiac disease are associated with increased intestinal permeability also known as “leaky gut” [12], [13]. Thus, gut leakiness in patients with a genetic susceptibility to PD may be a pivotal early step promoting a pro-inflammatory/oxidative environment contributing to the initiation and/or progression of the PD process. One particularly detrimental consequence of increased intestinal permeability is the translocation of bacteria (e.g., *E. Coli*) and bacterial products (e.g., lipopolysaccharide (LPS) also known as endotoxin) which creates a proinflammatory environment and increases the oxidative stress burden in the enteric nervous system. Indeed, it has been suggested that the GI tract might be a portal of entry for a putative PD pathogen, triggering pathological changes in the submucosal/myenteric neurons, which then spread through the vagus nerve to the medulla oblongata [14], [15]. From there, pathological changes may move rostrally, ultimately resulting in the clinically-defining motor symptoms of PD when there is extensive involvement in the middle portion of the disease at the level of the midbrain substantia nigra [5]. Thus, the involvement of the GI tract in PD is of great interest as a contributing factor to the development and progression of PD.

To date, intestinal permeability has not been investigated in patients with PD. Accordingly, we hypothesized that PD subjects might have increased intestinal permeability leading to increased exposure of intestinal neuronal tissue to bacterial derived pro-inflammatory products resulting in oxidative stress and neuronal pathological α-synuclein aggregates. To this end, the objectives of this study were to: i) assess intestinal permeability in subjects with newly diagnosed, untreated PD and compare the results to controls without a history of neurological disease or PD; ii) determine whether increased intestinal permeability (“leaky gut”) in PD subjects correlated with markers of bacterial translocation and endotoxin exposure either locally or systemically, and iii) determine whether gut leakiness is associated with mucosal oxidative stress and intestinal neuronal α-synuclein aggregates.

## Results

**Figure 1:**
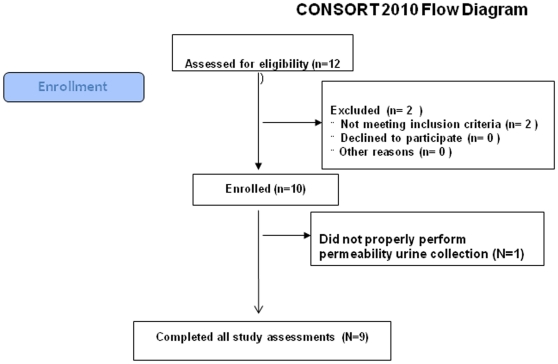
Consort 2010 flow diagram of this study. 12 research subjects were invited to participate in this study. Two were excluded because they failed to meet inclusion criteria (prohibited medications). One study subject incorrectly performed the 24-hour urine collection for intestinal permeability testing and his data were excluded from analysis. Nine study subjects completed all study assessments.

We studied 9 subjects (6 men) with PD (see flow diagram Fig. 1). Subject demographics and disease duration and bowel movement (BM) characteristics are shown in Table 1. Median age was 57. Median duration of PD from first symptom onset was 2 years. Median total Unified Parkinson's Disease Rating Scale (UPDRS) score was 24; median Hoehn & Yahr stage was 2. No patient took levodopa or dopamine agonists, although one took amantadine. Importantly, none of our PD or control subjects had symptoms of constipation. PD subjects were compared to ten age-matched control subjects (7 men), median age 49 with no signs or symptoms of neurological diseases or other chronic diseases (no PD vs. Controls statistical age difference by Mann-Whitney). No bleeding, fever, pain or other side effects of any procedures were detected in any subjects.

**Table 1:** Parkinson's Disease Subject Characteristics.

Age (Y)/Sex	Disease Duration (Y)	UPDRS Score	HY Stage	BM/week	Diarrhea	Constipation
75M	4	28	2	4	n	n
68F	0.5	24	2	14	n	n
66M	1	27	2	7	n	n
61M	1	18	2	7	n	n
57M	2	28	2	10	n	n
56F	1	15	1.5	10	n	n
55M	4	28	2	7	n	n
47M	8	18	2	10	n	n
46F	2	16	1	5	n	n
**Median 57**	**2**	**24**	**2**	**7**	**-**	**-**

### Parkinson's disease subjects have significantly increased whole gut permeability to sucralose

**Figure 2:**
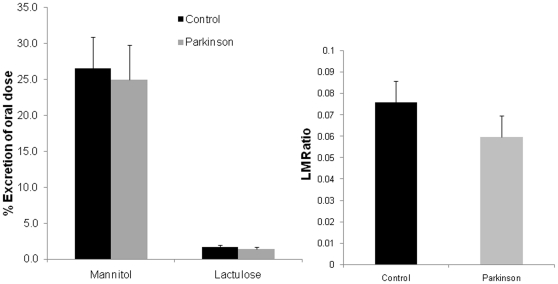
Intestinal permeability to lactulose and mannitol is similar in control and PD subjects. PD subjects and healthy controls were assessed for intestinal permeability using an oral sugar solution containing sucrose, lactulose, mannitol, and sucralose and GC analysis of 24 hour urine samples as described in Materials and Methods. Urinary lactulose and mannitol as well as the L/M ratio (all primarily measures of small intestinal permeability) were not significantly different in PD patients compared to controls. Data are presented as mean % urinary excretion of the oral dose in 24 hours ± SE. * p<0.05.

**Figure 3:**
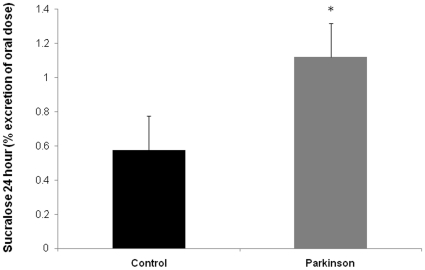
Intestinal permeability to sucralose is significantly greater in PD patients. PD subjects and healthy controls were assessed for intestinal permeability using an oral sugar solution containing sucrose, lactulose, mannitol, and sucralose and GC analysis of 24 hour urine samples as described in Materials and Methods. Urinary sucralose, a measure of whole intestine permeability, was significantly greater in PD subjects vs. healthy age matched controls. Data are presented as mean % urinary excretion of the oral dose in 24 hours ± SE. * p<0.05.

Twelve hour urinary lactulose or mannitol as well as the L/M ratio in subjects with PD were similar to the values obtained for controls (Fig. 2). In contrast, 24 hour urinary sucralose (marker of total intestinal permeability) was significantly increased in PD subjects (Fig. 3). As seen in Figure 3, the mean 24 h urinary sucralose (expressed as percent of oral dose) was significantly greater (about double versus controls) in PD subjects (1.12±0.1) compared to age matched controls (0.58±.1) (p<0.05).

### PD subjects show significant evidence of increased exposure to intestinal bacteria and bacterial endotoxin

**Figure 4:**
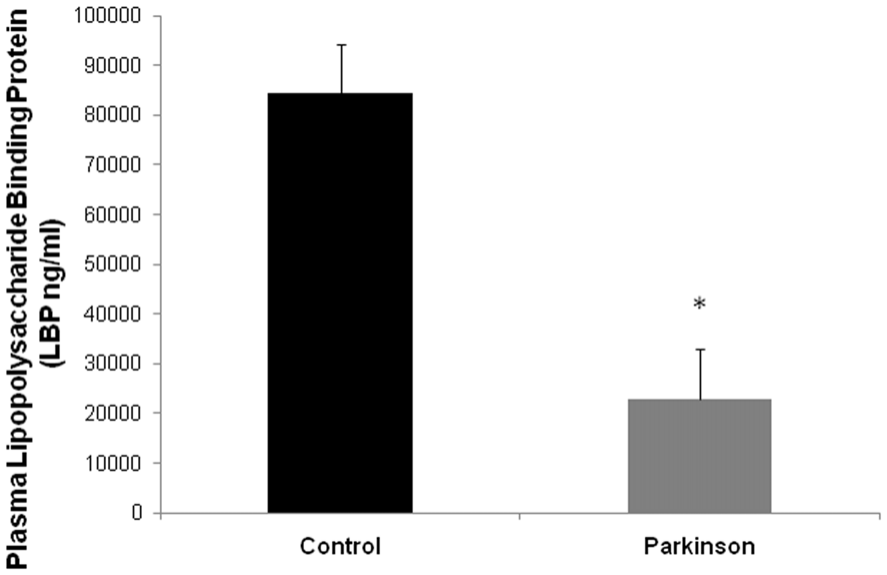
Plasma LBP is significantly lower in PD patients. Plasma levels of LPS binding protein (LBP), an indirect measure of systemic endotoxin exposure, were determined for PD subjects and healthy controls as described in Materials and Methods. Values for plasma LBP in PD subjects were significantly lower than in healthy controls. Data are presented as means (ng/ml) ± SE. *p<0.05.

To determine if the increased intestinal permeability observed in PD subjects was associated with increased translocation of intestinal bacterial products, we stained sigmoid mucosa slides with polyclonal Ab for the gram negative bacteria *E. Coli*. We found that there was significantly more intense staining of *E. coli* in both epithelial and lamina propria zones of sigmoid mucosa samples from patients with PD compared to controls (Table 2). Furthermore, there was a significant correlation (Pearson's r = 0.632; p<0.05) between intensity score for lamina propria zone staining of *E. coli* and urinary sucralose (intestinal permeability) in PD patients (Table 3). Thus, these results not only support the conclusion of the urinary sugar studies (i.e., PD subjects have increased colonic permeability), it also suggests that enhanced permeability in PD subjects may have potentially significant biological consequences resulting from increased exposure of neuronal tissues in mucosa and possibly sub-mucosal (lamina propria) parts of the colonic wall to bacterial products including endotoxin. As another index of intestinal permeability, systemic exposure to intestinal bacterial products was determined by measuring plasma LPS binding protein (LBP) [16], [17]. Lower levels of plasma LBP have been associated with increased exposure to gram negative bacteria [16], [18]. As shown in Fig. 4, PD subjects had a significantly lower mean level of plasma LBP compared to normal subjects (PD 22856±5540 ng/ml vs. Control 84291±31380 ng/ml). Other measures of systemic endotoxin exposure such as serum endotoxin, plasma IgG endocab (native Ab to LPS), and serum soluble CD14 were not different between the PD and control groups (Table 4). Taken together these plasma LBP and *E. coli* staining data support increased intestinal permeability to gram negative bacteria and bacterial products in PD subjects.

**Table 2:** *E. coli* Staining Scores for Intestinal Tissue.

Group	Score for Intensity of *E. coli* Staining			
	Lumen	Lamina Propria	Epithelial	Crypt
**Control**	2.5 (0–3)	0.5 (0–4)	1 (0–3)	0 (0–3)
**Parkinson**	3 (1–3)	2* (1–4)	2.5* (2–4)	3 (0–3)

*Correlation is significant at the 0.05 level (2-tailed).

Scores are shown as medians and (ranges).

**Table 3:** Spearman's r Correlation Values.

				Score for Intensity of *E. coli* Staining			
	Nitrotyrosine	Sucralose	PlasmaLBP	Lumen	Laminapropria	Epithelial	Crypt
**α-Synuclein**	0.911**	0.588*	−0.853**	−0.181	0.632*	0.330	0.133
**Nitrotyrosine**	NA	0.609*	−0.822*	−0.284	0.539*	0.312	0.266
**Sucralose**	0.609*	NA	−0.475	−0.128	0.672*	0.121	0.174

**Correlation is significant at the 0.01 level (2-tailed).

*Correlation is significant at the 0.05 level (2-tailed).

**Table 4:** Measures of Endotoxin Exposure.

	Endotoxin(EU/ml)	sCD14(ng/ml)	Endocab IgG(GMU/ml)	LBP(ng/ml)
**Controls**(mean ± SE)	0.821±208	2022±143	488±80	84291±31380
**Parkinson**(mean ± SE)	0.840±.127	2007±76	433±54	22856±5540
P value	0.71	0.88	0.68	*0.016

*PD vs. Control difference is significant at the 0.05 level (2-tailed).

### Increased intestinal biopsy staining for α-synuclein and 3-nitrotyrosine (3-NT) correlate significantly with intestinal hyperpermeability and E. coli staining in PD subjects

**Figure 5:**
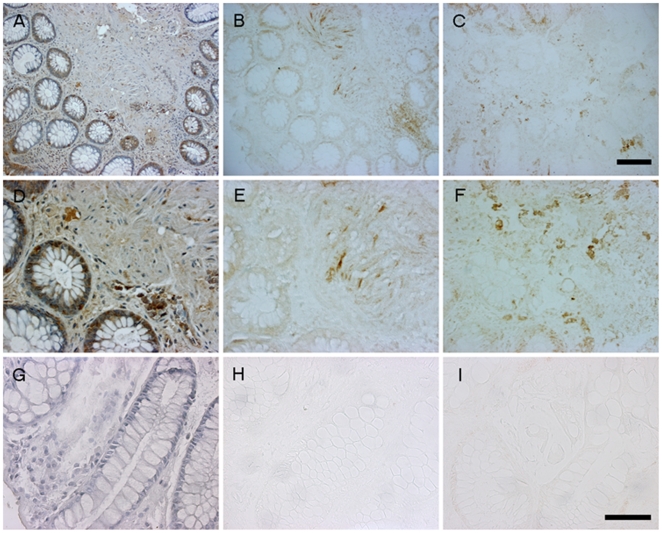
Immunohistochemical staining of intestinal biopsies for *E. coli*, α-synuclein, and 3-NT is significantly greater in PD subjects. Intestinal biopsies from PD subjects and healthy controls were formalin fixed, paraffin embedded, and then cut to 5 µm and processed as described in Materials and Methods for each respective antibody to either *E.coli* gram negative bacteria (A,D,G), α-synuclein (B,E,H) or 3-nitrotyrosine (3-NT)(C,F,I). Representative images are shown from a PD subject at 15× (A–C) and 40× (D–F) magnification using the same field of view and from a healthy control subject (G–I) at 40×. Staining data were analyzed by blinded observers and PD subjects were found to stain significantly greater for *E. coli*, α-synuclein, and 3-NT. Scale bars = A–C 50 µm, D–I 100 µm.

We next wished to examine if increased intestinal permeability to inflammatory bacterial products measured in PD subjects was correlated with intestinal markers of PD (e.g., α-synuclein) and inflammation/oxidative stress (e.g., nitrotyrosine). We have recently demonstrated that these PD subjects exhibit significantly increased intestinal staining for both α-synuclein and nitrotyrosine [19]. To further investigate the relationship between permeability to bacterial products, α-synuclein, and nitrotyrosine staining we re-stained intestinal biopsies from PD subjects and controls using antibodies to all three markers. Fig. 5 shows representative images from both a PD subject (A–F) as well as a control subject (G–I) using the same field of view from each respective tissue sample. Clearly, the tissue from the PD subject shows significantly greater staining for *E. coli*, α-synuclein and nitrotyrosine when compared to the control subject tissue staining. To determine whether increased gut leakiness and increased exposure to LPS are associated with oxidative stress (nitrotyrosine staining) and PD neuronal injury (α-synuclein staining) in these subjects, we determined Spearman's “r” correlation coefficients between urinary sucralose levels with the intensity staining scores of *E. coli* staining, α-synuclein staining and nitrotyrosine staining for all PD subjects and controls (Table 3). Very importantly, we found that increased intestinal permeability (i.e., urinary sucralose) and *E. coli* staining significantly correlated with α-synuclein staining in PD subjects but not in controls. Remarkably, increased urinary sucralose, *E. coli* staining, and α-synuclein staining also each significantly correlated with increased intestinal staining for nitrotyrosine in PD subjects but not in healthy controls.

## Discussion

GI symptoms such as constipation and bloating are the most common non-motor symptoms in PD patients [20]. It is generally believed that these symptoms are a consequence of PD and are the result of intestinal motility disorders and the associated intestinal bacterial overgrowth. However, over the last decade there has been mounting evidence that supports a role for the GI tract and the enteric nervous system (ENS) in the pathogenesis of PD [7], [15]. Two early studies found the first GI Lewy bodies (LBs) in the esophagus and colon in PD patients [21], [22]. Later studies by a third group substantiated these early findings of LB pathology in both the submucosal plexis (SMP) and myenteric plexis (MP) in the ENS of PD patients [23], [24], [25]. Recently, Lewy bodies were found in colonic biopsies from 4 out of 5 [26] and 21 out of 29 [27] PD patients, but in none of the controls. It is notable that all of these prior studies used later stage patients. The similarity of our findings in early untreated patients suggests that this pathology is unrelated to drug treatment and supports the concept for GI-related parameters to serve as a biomarker for the disease.

None of our subjects complained of constipation and the frequency of bowel movement in all subjects were within normal range. These data are now included in Table 1. In fact, we designed the study as such to minimize the potential confounding effects of constipation due to longstanding Parkinson's disease and included only newly diagnosed PD patients. Thus, it is unlikely that constipation played a role in any of our findings. Although we did not directly study small bowel bacterial overgrowth, none of the patients complained of bloating and there is no known physiological reason for small bowel bacterial overgrowth to cause disruption of colonic permeability. Of course changes in colonic microbiota could play a critical role in changes in colonic permeability in PD patients because there is compelling evidence of cross talk occurring between the colonic microbiota and intestinal epithelial cells. Indeed, part of our broad hypothesis is that changes in colonic microbiota could play a mechanistic role in our observed intestinal hyperpermeability and further studies are needed to interrogate intestinal microbiota in patients with PD.

While compelling, these observations do not necessarily suggest a causal role for the intestines in the pathogenesis of PD and could simply indicate widespread neuronal damage resulting from PD. However, these findings by others, as well as by Braak and colleagues, led to the proposal [14], [15] that the ENS may be a route by which a toxin or pathogen initiates the progression of PD over a period of many years. In support, a pathway of α-synuclein expressing neurons from the gut to the CNS was recently shown [28]. A recent publication from our group has shown α-synuclein staining positive sigmoid mucosal neuronal tissue in all 9 of the early untreated PD patients examined in this present study[29], while none of the control samples exhibited substantial staining indicating these processes occur early on in the pathogenesis of PD. None the less, these observations still do not prove that PD pathology begins in the ENS and they may simply represent progression of PD pathology that could explain the high frequency of GI symptoms in PD patients [7]. Further longitudinal studies are needed to determine whether α-synuclein staining in the intestinal mucosa is present in PD patients years prior to onset of clinical neurological evidence of PD.

Regardless of whether intestinal α-synuclein aggregates are the primary or secondary events in PD, in order to better understand its role in PD, we need to determine the mechanism of their development. It is now widely believed that α-synuclein aggregates are the consequence of oxidative injury to neurons [4]. It is also well established that inflammation and pro-inflammatory cytokines like IL-1β, IL-6 and TNF-α are one major source of tissue oxidative stress [30]. Indeed, neuroinflammation is present within the brain of early PD patients [31] and considerable evidence supports a role for neuroinflammation in PD pathogenesis [30], [32].

Thus, it is a reasonable and plausible hypothesis that ENS α-synuclein aggregates are the consequence of a local pro-inflammatory milieu. Braak and colleagues propose that toxin and/or pathogen presence in the intestinal lumen may trigger neuroinflammation. However, it should be noted that the presence of toxins and/or pathogens might not necessarily supply the required trigger because even healthy subjects have a large amount of luminal proinflammatory products resulting from normal intestinal microbiota including endotoxins. It is more compelling to consider that increased intestinal permeability (leaky gut) and translocation of these substances is the critical factor for intestinal neuronal oxidative injury. Several lines of evidence strongly suggest that endotoxins are plausible neuroinflammatory triggers in PD. First, endotoxin (LPS) has been suggested as one environmental trigger of PD [33]. Second, LPS administration either systemically or directly into the CNS is a widely used animal model of PD. For example, mice given a single dose of LPS systemically [34] or prenatally [35] develop CNS inflammation including selective loss of DA neurons in the substantia nigra (SN) [34]. In the present study we showed evidence of excessive endotoxin exposure in PD patients because serum LPS binding proteins were significantly lower in PD patients compared to controls. Generally, lower levels of LBP have been shown to support increased proinflammatory signaling and cytokine production by endotoxin [16]. To the best of our knowledge, our finding is the first to show increased bacterial/endotoxin exposures in PD. It should be noted that we did not find significant increase in serum endotoxin in PD patients. We believe that our findings of significant changes in LBP and positive staining of *E.coli* antigen in the colonic biopsies strongly suggest that our observed increased intestinal permeability in PD has significant biological consequence resulting in exposure of intestinal mucosa and the mucosal immune system to the luminal bacteria products. However, the translocation of luminal bacteria products could be limited to the colonic submucosa leading to oxidative injury to the colonic mucosa neurons and aggregation of α-synuclein. Another possibility is that leaked endotoxin could reach the portal systemic circulation but is cleared by the liver and thus a high level of endotoxin was not observed in our patients. Regardless, even if leaked endotoxin cannot reach the circulation, it still can play a central role in production of α-synuclein aggregates in the colon and pathogenesis of PD. Endotoxins (or gram negative bacteria such as *E. coli*) can bind to and thus be trapped in the intestinal mucosa and not reach the systemic circulation. Positive *E. coli* staining in the sigmoid mucosa of our PD patients not only further supports the conclusion of increased permeation of endotoxins across intestinal epithelial cells, it also supports the possibility that endotoxins become trapped in the intestinal mucosa. It should also be noted that endotoxins might be directly involved in initiation and/or progression of neuroinflammatory injury in both the ENS and CNS in PD without systemic endotoxemia [36]. Further studies are needed to evaluate whether there is cell loss in the enteric nervous system associated with α-synuclein aggregates in our PD subjects

How does endotoxin promote neuroinflammation? One possibility is that endotoxins stimulate the enteric immune response either directly or via glial cells to promote local oxidative stress leading to α-synuclein misfolding, aggregation, and subsequent neuronal damage in the ENS in individuals genetically susceptible for PD. Indeed, we have shown an increased nitrotyrosine staining indicating increased oxidative stress in the sigmoid mucosa of PD patients and this increase correlated with plasma LBP and intensity of intestinal *E. coli* staining. Our finding that serum sCD14 levels in PD patients was not significantly increased suggests that macrophages are not involved in endotoxin-induced neuroinflammation because activation of macrophages by endotoxin through ligation with TLR4 results in shedding of sCD14 and increased levels of sCD14 in the blood [37]. However, it is possible that shedding of sCD14 in the intestinal mucosa is not reflected in the serum measurement. It should also be noted that endotoxins can trigger immune-inflammatory pathways via TLR4 ligation without requirement of CD14 in non-macrophage immune cells [38].

If indeed endotoxins cause neuroinflammation then why does this occur only in individuals susceptible for PD? First, neuronal tissues in those susceptible to PD may be more sensitive to the endotoxin effects. There are several experimental findings to support this possibility. Rodent nigral neurons have been shown to be more vulnerable than either hippocampal or cortical neurons to LPS-induced degeneration *in vivo* and *in vitro*, and this was shown to be due to the higher number of activated microglia per unit area in the SNpc compared with the other brain regions [39]. Inherent processing of α-synuclein may also play a role. Transgenic mice overexpressing human A53T mutant α-synuclein were given LPS systemically (i.e., i.p. injection). Only the transgenic mice, and not controls, developed sustained neuroinflammation including Lewy bodies staining of nigral neurons [40]. This supports a synergistic interaction between defective α-synuclein processing and endotoxin exposure in promoting PD-related nerve pathology.

Second, endotoxin-mediated neuronal damage might not be limited to PD. For example, normal human rectal tissue biopsies incubated with LPS exhibit increased enteric glial cell oxidative stress burden (i.e., iNOS production) [41], a possible source for the observed nitrotyrosine staining in our subjects. Furthermore, co-culture of rat enteric neurons and enteroglial cells stimulated with LPS results in increased glial cell production of proinflammatory cytokine IL-1β [42]. It is therefore not surprising that endotoxemia has been reported in other neurological disorders like autism, ALS, and depression [43], [44] [45].

Another question that needs to be answered is what is the cause of increased endotoxin exposure in PD? One obvious mechanism is increased intestinal permeation to endotoxin (i.e., gut leakiness). We show here that PD patients have a “leaky gut”. PD patients not only had marked and significant increased urinary sucralose, they also had increased intensity of *E. coli* staining in the deep zone of the sigmoid mucosa. Thus, PD patients appear to have a hyperpermeable intestinal epithelium and this may have the important biological consequence of a significantly increased exposure to endotoxins, oxidative stress, and neuronal injury to the ENS. The increase in urinary sucralose with no significant change in urinary lactulose or L/M ratio in PD patients strongly suggests that the site of enhanced leakiness is the colon. To our knowledge, intestinal permeability has previously been evaluated in PD subjects in only one other study [46]. In that study they found PD patients exhibited decreased absorption of mannitol resulting in an increased L/M ratio. However sucralose absorption was not tested as we have shown in our study and very significantly the median age of those patients was 73.9 y/o (vs. 57 y/o in our study) and all had responded to L-dopa therapy (vs. no therapy in our study). Also, no intestinal biopsy staining for α-synuclein or markers of endotoxin was carried out. Thus we are the first to demonstrate increased intestinal permeability and its significant association with increased intestinal exposure to endotoxin, intestinal oxidative stress, and intestinal α-synuclein aggregation in early, untreated PD patients.

In summary, our study presents data for a new model for PD pathogenesis involving increased intestinal permeability to proinflammatory bacteria and bacterial products such as endotoxins (i.e., LPS). This model provides for an easily obtained, inexpensive biomarker that may be useful in identifying those individuals with premotor PD that will get classic PD signs and symptoms later in life. Our data demonstrate that increased intestinal permeability strongly correlates with markers of increased exposure to endotoxin and with a marker indicating increased oxidative stress burden in the intestine, which together may be responsible for the abnormal accumulation of α-synuclein in enteric neurons we also observe as have other studies. Such increased bacterial endotoxin may therefore initiate a cascade of proinflammatory events promoting PD in genetically susceptible individuals. We acknowledge that our cross sectional observational study cannot establish a causal relationship between increased permeability and PD pathophysiology and that further interventional and animal studies are needed to determine whether gut leakiness and α-synuclein deposition in the colon have a causal role in the pathogenesis of PD. These studies will be needed to determine whether increased intestinal permeability plays a causative role in PD initiation or is a consequence of PD pathogenesis. At the least, such a mechanism for increased exposure of the ENS to intestinal endotoxin may fuel the progression of PD in susceptible subjects.

## Materials and Methods

### Study Subjects and Ethics Statement

The protocol for this trial and supporting CONSORT checklist are available as supporting information; see Checklist S1 and Protocol S1. The study was reviewed and approved by the Institutional Review Board at Rush University Medical Center, and registered with Clinicaltrials.gov (NCT NCT01155492, “Increased Gut Permeability to Lipopolysaccharides (LPS) in Parkinson's Disease”). No deviations from the approved trial protocol were made in this study. All subjects provided written informed consent. We recruited subjects with clinically diagnosed Parkinson's disease not yet requiring dopaminergic therapy. None of our PD subjects exhibited symptoms of constipation. Constipation was defined as fewer than 3 BM/week or if the subject complained of constipation. Men and women who met United Kingdom Parkinson Disease Research Society brain bank criteria for PD, Hoehn & Yahr stage 1–2.5 [47] were included. Subjects were excluded based on the following: atypical or secondary Parkinsonism, any known organic gastrointestinal disease, use of drugs affecting gastrointestinal motility, anti-inflammatory agents, and chronic diuretic use. Control subjects were of similar age and gender who had no GI or neurological symptoms or signs (determined by examination by a board certified neurologist with expertise in movement disorders) and were not taking regular medication or anti-inflammatory agents.

### Intestinal permeability measurement (primary outcome)

One way to assess intestinal permeability is by administration of oral sugars (i.e., mannitol, lactulose, sucrose, and sucralose) and analysis of subsequent sugar excretion in urine. Since these sugars are not significantly metabolized in the body after absorption from the intestine, excretion into the urine reflects intestinal permeability [48]. Passageways (“pores”) formed by tight junctions between GI epithelial cells range in size from 4–60 Å and differentially allow the passage of molecules. These characteristics in conjunction with attributes of each sugar allow for determination of regional differences in GI permeability. Small molecules such as mannitol traverse pores of all sizes, while larger molecules, such as lactulose, can only pass through larger pores [11], [48]. Sucrose is rapidly degraded after leaving the stomach, so increased sucrose excretion reflects gastric permeability and sucralose is absorbed through large pores in the small and large intestine. Increased urinary sucrose, lactulose/mannitol ratio and sucralose reflect gastroduodenal, small intestinal and total gut (small bowel and large bowel) hyperpermeability, respectively [48], [49]. Increased sucralose excretion in conjunction with normal lactulose/mannitol ratio might reflect increased large intestinal (colonic) permeability [48]. The rationale for using urinary sucralose as a reliable marker of total gut permeability is that not only is sucralose relatively uniformly absorbed in both small and large intestine it is also available in the lumen of the colon for absorption because, unlike lactulose and mannitol, it cannot be metabolized and consumed by colonic bacteria.

Subjects fasted overnight and subsequently ingested a sugar mixture containing 2 grams mannitol, 7.5 grams lactulose, 40 gm sucrose, and 1 gram sucralose at 6AM, then collected 2 sequential 12-hour urine samples. Urine was analyzed for sugar content using gas chromatography (GC) techniques. Measurement of urinary sugars using GC is used to calculate intestinal permeability and is expressed as percent oral dose excreted in the urine. We have recently revised our method which briefly involves conversion of the relevant sugars to their alditol acetate form rather than our previous method of N-Trimethylsilylimidazole (TMSI) derivatization and find it is a more sensitive method to detect the sugars. This is thus a modification of our methods that we have previously published [49], [50].

### Plasma and serum measures of endotoxin exposure (secondary outcome)

Endotoxin was measured in serum by Limulus Amebocyte Lysate QCL-1000 from Lonza (catalogue # 50-647U)(EU/ml). Serum or plasma samples were diluted at 1∶5 ratio with LAL reagent water. Endotoxin IgG core antibodies were measured in plasma using an ELISA kit from Cell Sciences Inc (catalogue # HK504). Units are expressed as GMU/ml which are IgG standard median units based on medians of ranges of 1000 healthy adults by the manufacturer. Soluble CD14 (sCD14) (ng/ml) was measured in serum by R&D systems quantikine immunoassay (catalogue # DC140). Lipopolysaccharide binding protein (LBP)(ng/ml) was measured in plasma using an ELISA kit from Cell Sciences Inc (catalogue # HK315).

### Immunohistochemistry staining for E. coli in intestinal biopsies

Limited unprepped flexible sigmoidoscopy to the distal sigmoid at around 20 cm from the anal verge was performed in control and PD subjects by one of the authors (AK). No sedation was required for the procedure, the duration of which was less than 10 minutes. No bleeding, fever, or other side effects were observed or reported in any subject. Six cold biopsies were obtained from normal appearing sigmoid colon using biopsy forceps. Samples were snap frozen in liquid nitrogen at the time of collection. Biopsy specimens used for staining were fixed in formalin and embedded in paraffin. 5 µm thick sections were cut for *E. coli*, α-synuclein, and 3-nitrotyrosine (3-NT) staining analysis. Immunohistochemistry for rabbit polyclonal anti-*Escherichia coli* (Cat. # B0357; Dako) was performed using a biotin-free polymer approach (Rabbit Prolink-1, Golden Bridge International, Inc.) on 5-µm tissue sections mounted on glass slides, which were dewaxed and rehydrated with double-distilled H_2_O. Antigen retrieval was performed by heating sections in 1× DIVA Decloaker reagent (Biocare Medical) in a pressure cooker set at 121°C for 30 sec. Slides were stained with optimal conditions determined empirically on an IntelliPATH autostainer (Biocare Medical) that consisted of a blocking step using blocking buffer (TBS with 0.05% Tween-20 and 0.5% casein) for 10 min and an endogenous peroxidase block using 1.5% (v/v) H_2_O_2_ in TBS (pH 7.4) for 10 min. Primary Abs were diluted in blocking buffer and incubated for 1 h at room temperature or overnight at 4°C. Tissue sections were washed, and the Rabbit Prolink-1 staining system (Golden Bridge International, Inc) was applied for 30 min at room temperature. Sections were developed with Impact™ 3,3′-diaminobenzidine (Vector Laboratories), counterstained with Hematoxylin and mounted in Permount (Fisher Scientific). All stained slides were scanned at high magnification (200×) using the ScanScope CS System (Aperio Technologies, Inc.) yielding high-resolution data for the entire tissue section. Representative high magnification (200×) images were acquired from these whole tissue scans for scoring.

Using *E. Coli* IHC as a measure of intestinal permeability is an approach which has recently been used successfully to assess intestinal leakiness to bacterial contents in HIV patients [51]. Staining was quantitated by blinded scoring on a scale of 0–4 with 4 as the highest in four separate intestinal tissue zones: Lumen (positive control), epithelium, lamina propria (LP), and crypts.

### Immunohistochemistry staining for α-synuclein and nitrotyrosine in intestinal biopsies

Sections were processed using an antigen-retrieval procedure. Briefly, the sections were treated with 88% formic acid for 20 min followed by citric acid (pH = 6.0, at boiling temp) treatment for 20 min, then kept outside at 25°C and processed through three washes of distilled water and three washes of phosphate buffer solution (PBS). Sections were incubated in 3% normal horse serum and 2% bovine serum albumin for an hour, then incubated in the primary anti-α-synuclein (mouse anti-alpha synuclein, 1∶500, Invitrogen) or 3-NT (Millipore Inc; 1∶500) antibody for 48 hrs at 4°C. The sections were washed, then sequentially incubated in the secondary antibody (biotinylated horse anti-mouse, 1∶200, Vector Laboratories), phosphate buffer saline washes, and the ABC solution (ABC kit, Vectastatin Elite, cat # PK-6100). The stain was completed in a chromogen solution containing 0.05% 3′3 diaminobenzidine and 0.005% hydrogen peroxide. After stain development, the sections were dried overnight, then coverslipped with cytoseal (23244257, Fisher). The same procedure was followed for brain slices including the substantia nigra which was used as a positive control (not shown). Negative control sections substituted an irrelevant IgG for primary antibody.

All immunostained slides were reviewed and rated for presence and intensity of α-synuclein and 3-NT staining by a blinded rater (HD) according to a 5 point scale with 0 = no immunostaining and 4 = very intense immunostaining.

### Statistical analysis

Unless otherwise noted, Data are presented as means ± SE. For non parametric analyses of two groups, we used Mann-Whitney U; (Figs. 2, 3, and 4 and Table 4).Correlational analyses were done using the Spearman test for nonparametric analysis (Table 3). For *E. coli* staining scores are shown as medians with the range (Table 2). *P* values<.05 were deemed statistically significant. All analysis except power analysis was performed using the SPSS statistical software (IBM, Armonk, NY). For power analysis intestinal permeability sucralose measurements were used. The means for sucralose percent urinary excretion of oral dose ± SE were 0.58±0.1, 1.12±0.1 in control and treatment groups respectively. For N = 10 in control and N = 9 in the PD group, with the alpha level at 0.05, a one sided test was applied and the power for our study is 0.80708 (81%). All power calculations were done in PASS (PASS 2008. NCSS, LLC. Kaysville, Utah).

## Supporting Information

Checklist S1 **The CONSORT checklist was prepared and references specific text in the study that fulfills the checklist requirements.**
(DOC)

Protocol S1 **A flow diagram illustrating the clinical study protocol described in this supporting document is shown as** **Figure 1** **.**
(DOC)
