# Supplementary material for: Increased Intestinal Permeability Correlates with Sigmoid Mucosa alpha-Synuclein Staining and Endotoxin Exposure Markers in Early Parkinson's Disease
Source: PLoS One. 2011 Dec 1;6(12):e28032. doi: 10.1371/journal.pone.0028032 (PMC3228722; doi:10.1371/journal.pone.0028032)
Supplement: Supplementary file 2 [file pone.0028032.s002.doc]

Clinical Protocol S1

Study Title:
Increased gut permeability to lipopolysaccharides (LPS) in Parkinson's Disease

Investigators:
Kathleen M. Shannon, M.D.; Department of Neurological Sciences; Rush University Medical Center; 1725 W. Harrison Street; Chicago, IL 60612

Ece Mutlu, M.D.; Department of Gastroenterology; Rush University Medical Center; 1725 W. Harrison Street; Suite 755; Chicago, IL 60612

Jeffrey H. Kordower, PhD; Research Center for Brain Repair, Rush University Medical Center, 1735 West Harrison Street, Cohn Research Bldg, 3rd Floor, Chicago Illinois 60612

Background:
Parkinson's disease
Parkinson's disease is the second most common neurodegenerative disease, affecting about 1 million Americans. Clinically characterized by tremor, rigidity, bradykinesia and disordered gait and balance, its pathological basis is neuronal loss subsequent to intracellular α-synuclein aggregation. While the focus has been on motor dysfunction and degeneration of central nervous system dopaminergic neurons, more recent evidence highlights non-motor, non-dopaminergic symptoms such as constipation, orthostatic hypotension, sleep and affective disorders (1-4)} and supports the concept that the peripheral and central nervous system and multiple neurochemicals are involved. (5)

The cause of PD is unknown. Current hypotheses on the etiology of Parkinson's disease posit both genetic and environmental factors. Mutations in genes involved in α-synuclein synthesis, ubiquitin-dependent proteasomal proteolysis, and in the regulation of neuronal process morphology have been identified in research subjects with dominantly or recessively inherited familial parkinsonism and even in sporadic Parkinson's disease, but these monogenic forms of Parkinson's disease are extremely rare. (6) Epidemiological studies suggest the risk of Parkinson's disease is associated with certain occupations (medicine, dentistry and teaching) and occupational (agriculture) and environmental (well-water drinking) exposures, but no common environmental trigger has yet been established. One hypothesis about environmental contributors to the etiology of Parkinson's disease is that these substances may cause Parkinson's disease by

inciting inflammatory mechanisms (neuroinflammation). Microglia, the resident immune cells of the brain, represent the first line of response to immunologic challenges and a play a primary role in neuroinflammation. The substantia nigra, a site of profound degeneration in Parkinson's disease, is an area that is particularly rich in microglia, making it especially vulnerable to neuroinflammation. A role of neuroinflammation in Parkinson's disease is supported by postmortem studies demonstrating elevated levels of proinflammatory factors (complement proteins, cytokines, TNF-α, etc.) in parkinsonian brain tissue and cerebrospinal fluid. Moreover, exposure to lipopolysaccharide causes a neuroinflammatory model of Parkinson's disease in experimental animals. (7)

LPS is a large molecule, an endotoxin, that is a major component of the outer membrane of gram-negative bacteria. LPS binds the CD14/TLR4/MD2 receptor complex, promoting the secretion of inflammatory cytokines. The primary source of human exposure to LPS is through the intestinal tract. There may be two main mechanisms through which there could be increased LPS in Parkinson's patients. One would be a defect in the intestinal barrier function that increases exposure to the LPS that is already produced in the GI tract. The other could be an alteration in the types of bacteria in GI tract that lead to an increased production of LPS. The aims of this proposal (shown below) are to study both of these potential mechanisms.

Multiple system atrophy is another neurodegenerative disorder with parkinsonian symptoms. Similar to Parkinson's disease, α-synuclein aggregates have been found in Multiple System Atrophy, but the aggregates affect astrocytes rather than neurons. It is not known why synuclein aggregates in different cells in the 2 conditions.

Gastrointestinal microbiota

Three main domains of life make up the phylogenetic tree of life: Bacteria, Archaea and Eucaryota (Fig.1). Human beings belong to eucaryota, but are super organisms that represent a fusion of eukaryotic cells and the bacteria and archaea that reside in and over the body. It has been estimated that up to 100 trillion micro-organisms reside in or on the human body. The majority of human body-associated microbiota reside in the gastrointestinal (GI) tract, which represents the most dense and least diverse ecosystem known. (8) Over 94% of the 400-1000 microbial species in the human gut are bacteria. To date, the bacteria and archaea have been cloned and sequenced from colonic tissue

samples and feces in three human beings. (8) Results demonstrate that GI bacteria belong to 8 out of 55 divisions and 5 of these are rare. The majority belong to two or three divisions: the Bacteroidetes and Firmicutes phyla that make up 30% each; less often to Proteobacteria are seen. (9) The GI microbiota can be highly variable in species and strains of individual bacteria. The current state of our knowledge indicates that GI microbiota are passed on from the mother to their infants, likely through exposure to the mothers' gut/perineal area at the time of vaginal delivery; that the composition of the GI microbiota is established by age 2 (10) and thereafter remains fairly stable through the majority of life. Large scale studies examining the gut microbiota have not been done primarily due to lack of methods to study these until recently. Compared to over 20,000 genes found in the human genome, the bacterial genomes (collectively known as the microbiome) can rapidly evolve under the pressure of changing environmental factors through non-replicative and replicative mechanisms that can be as short as minutes, compared to the lengthy human life. The sequence, activity, and evolution of majority of the microbial genes are relatively unknown, and has been the impetus for a second "human genome project," making microbiota and especially the GI tract microbiota, one of the biggest new frontier in health research.

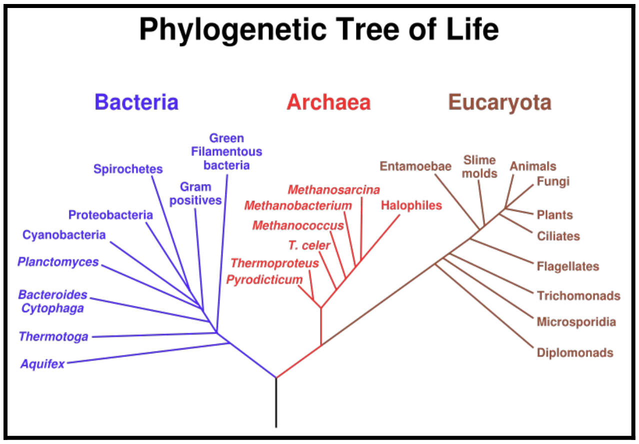

Figure 1

What is known about gastrointestinal tract bacteria and intestinal permeability in Parkinson's Disease?

Little is known about intestinal permeability and Parkinson's disease. A single limited study of 15 patients has not found an increased generalized non-carrier dependent small intestinal permeability (11) This suggests that the source of LPS exposure in Parkinson's disease may be altered colonic microbiota rather that generalized increases in permeability. Alternatively the source of increased permeability could be the proximal small intestine or the colon. To answer these questions, as planned in this current proposal, further assessments with multiple permeability probes (the multiple sugars used in this study) and further assessment of the composition of the colonic bacterial microbiota are needed.

Study objective:
We hypothesize that increased intestinal permeability to lipopolysaccharide or other bacterial toxins may predispose to the development of Parkinson's disease. Therefore, we will attempt to:

1. Determine the types of bacteria in the GI tract of Parkinson's disease patients and patients with multiple system atrophy.
2. Determine gut leakiness in Parkinson's disease patients and patients with multiple system atrophy.
3. Correlate the above to clinical parameters relevant to Parkinson's disease and multiple system atrophy.

in patients with early Parkinson's disease who are not yet taking antiparkinsonian drugs.

Methods:

Study subjects

Parkinson's disease group*:* 40 subjects with Parkinson's disease of various stages (stage 2-4), 10 subjects with Multiple System Atrophy , and 10 concurrent control subjects will be recruited to participate in the study by Dr. Shannon from the Parkinson's disease clinic. Controls will be recruited from spouses of Parkinson's disease patients.

Inclusion criteria:
1. Clinical diagnosis of Parkinson's disease by United Kingdom Parkinson Disease Society Brain Bank criteria or Multiple System Atrophy by Concensus criteria will be recruited.
2. Hoehn & Yahr 2-4
3. Parkinson's disease Symptomatic treatment for Parkinson's disease or Multiple System Atrophy will be allowed.

Exclusion criteria:
1. Occupation expected to change intestinal flora pattern (e.g. sanitation worker)
2. Treatment with medications that may induce parkinsonism (metoclopramide, typical or atypical antipsychotic agents)

3. Treatment within 12 weeks with systemic antibiotics
4. Known diagnosis of inflammatory bowel disease
5. Symptomatic organic GI disease other than hemorrhoids and hiatal hernia or abdominal surgeries for symptomatic GI disease such as bowel resection, diverticular surgery, colostomy, etc (subjects with a history of an appendectomy or cholesytectomy for benign disease more than 5 years prior to presentation are allowed).
6. Symptomatic functional GI disease that significantly impairs intestinal motility such as scleroderma or use of GI motility drugs
7. Acute illness requiring immediate hospitalization
8. Pre-existent organ failure or comorbidities as these may change GI flora:
    a) Liver disease (cirrhosis or persistently abnormal AST or ALT that are 2X> normal);
    b) Kidney disease (creatinine>2.0 mg/dL);
    c) Uncontrolled psychiatric illness;
    d) Clinically important lung disease or heart failure;
    e) HIV disease;
    f) Alcoholism, unreliable drinking history; or consumption of alcohol more than 3 times a week or binge drinking or drinking more than or equal to 3 drinks per occasion;
    g) Transplant recipients;
    h) Diabetes;
    i) Clinically significant dehydration or clinically detectable ascites or peripheral edema or cardiac failure
9. Presence of short bowel syndrome or severe malnutrition with ideal body weight < or = 90% or
10. Obesity with BMI >30;
11. Estimated survival <1 year and Karnofsky performance status <50%;
12. Daily use of anticoagulation or antiplatelet medications;
13. Use of immunosuppressive medications within 3 months of enrollment
14. NSAID use within 3 weeks of enrollment.
15. Chronic use of diuretics

<u>Control group</u>:
10 subjects who do not have Parkinson’s disease matched to subjects with Parkinson’s disease for age and gender will be recruited to participate in the study by Dr. Shannon among spouses or unrelated family members of Parkinson’s disease and Multiple System Atrophy patients.

Inclusion criteria

1. No evidence of GI symptoms other than minor hematochezia attributable to hemorrhoids
2. No evidence of symptoms of Parkinson's disease or Multiple System Atrophy
3. Matching in age and gender to the Parkinson's disease or Multiple System Atrophy patients.

Exclusion criteria:

1. Presence of Parkinson's disease, Multiple System Atrophy or its symptoms
2. All other criteria from 1-15 same as the Parkinson's group

Study procedures

1. <u>Parkinson's disease and Multiple System Atrophy subjects*:*</u>

Subjects that fulfill the inclusion and exclusion criteria will be recruited by Dr. Shannon and her clinical coordinator. Informed consent will be obtained from these subjects by Dr. Shannon and her coordinator. There will be at most four study visits- one for enrollment and clinical data collection (expected duration 1 hour), the second for urine drop off after collection (expected duration 5 minutes), the third for a limited unprepped flexible sigmoidoscopy (expected duration 10 minutes). The second and third visits may be combined for those patients who wish to do so. The last and fourth visit will be a month after the sigmoidoscopy to collect rectal swabs and patients will then also drop off a stool sample collected at home (expected duration 20 minutes).

a) clinical data:

i. Age
ii. Gender
iii. Race/ethnicity
iv. Parkinson related data:
   - age at Parkinson's disease or Multiple System Atrophy onset
   - Hoehn & Yahr stage (see Appendix)
   - Unified Parkinson Disease Rating Scale (see Appendix)
   - MMSE (see Appendix)

v. GI symptoms (see Appendix)

Subjects with GI symptoms that are rated major or severe on the GI symptom checklist and/or observed by Dr. Shannon to be clinically significant requiring further GI workup such as major rectal

bleeding, major abdominal pain will be excluded and referred for a clinical GI evaluation. Those subjects with minor symptoms such as constipation managed by over the counter laxatives or by fiber will be included and kept in the study.

b) CBC and CMP for inclusion and exclusion criteria

c) A blood sample for cytokine and endotoxin levels will be obtained andstored in the GI research freezers at -70 C for future analysis.

d) Sample collection for intestinal permeability:

The subjects will be told to given a collection of the following sugars (2 gm of mannitol, 7.5 gm of lactulose, and 40 mg of sucrose) to consume in 150 cc of tap water and 1 gm of sucralose (in 4 capsules) to swallow at 6 PM . After consumption of the sugars, the subjects will be asked to collect their urine for 24 hours. The subject will be given two containers: one for the first 12 hours of collection and a second for the second 12 hours of collection. The subjects will be asked to refrain from the consumption of the following foods that contain sugars for 24 hours during the urine collection: dairy (such as milk, cream, yogurt, cheese, ice cream, pudding) and sweets (cookies, candy, cake, chocolate, any kind of breakfast syrup). The subject will be provided with ice packs to keep their urine cold during the collection process, and asked to bring the collected urine within 24 hours after collection to the GI section clinical offices located at suite 207. One of the GI section research assistants (Sam Bracamonte, Lamis Eli, Delia Daian, Hauze Zhang, Cynthia Vann) will be responsible for receiving the sample from the subjects and will aliquot and store the samples in the GI section research freezers until analysis at -70 C.

e) Sample collection for colonic micribiota composition:

To assess colonic microbiota subjects will undergo a limited unprepped flexible sigmoidoscopy to the distal sigmoid at around 20 cm from the anal verge. The subjects will not be sedated. The standard flexible sigmoidoscope will be inserted to the rectum and will be advanced to 20 cm and six cold biopsies will be obtained from normal appearing sigmoid colon. Also a stool sample and rectal swabs will be obtained. The procedure lasts about 5 minutes. Subjects who are seen to have a significant lesion such as a polyp or tumor or ulcer or colitis will be excluded from the study and will be referred for clinical care for a full colonoscopy to a physician of their choice. All samples will be immediately snap frozen at the time of collection in liquid nitrogen and were stored in a – 70 ^{o}C freezer until analysis for colonic microbiota.

f) Follow-up visit:

Subjects will return in 1 month and will be asked to bring a stool sample.

At that time, Drs. Keshavarzian and Mutlu will also obtain two rectal swabs with 0.5 cm insertion of a sterile rectal swab into the rectum.

2. Control subjects:

Age- and gender-matched subjects that fulfill the inclusion and exclusion criteria for this group will be recruited by Dr. Shannon from Parkinson's disease or Multiple System Atrophy spouses or unrelated family members.

After consent, the same exact types of data (clinical data; urine sample for permeability; biopsy and stool samples and rectal swabs for colonic microbiota analysis) will be collected from the control subjects as the study group of patients with Parkinson's disease. Additionally, this control group will be screened for the presence of Parkinson's disease by administration of a checklist for Parkinsonian symptoms. If there are symptoms or signs of the disease upon evaluation of this list and the clinical evaluation of Drs. Keshavarzian, subjects will be excluded and will be referred to neurology for clinical evaluation.

C. Laboratory procedures and analyses:

1. Intestinal permeability: Gas chromatography will be performed using a Hewlett Packard instrument (HP6890N Palo Alto, CA) equipped with a Flame Ionization Detector (FID). We will use DB-225 capillary column (J&W, Folsom, CA), which is 30m X 250 μm I.D. column, with a 0.25 μm film thickness. The detector temperature will be 300˚C and the injector temperature will be 250˚C. The initial column temperature of 170˚C will be held for 8 min and then increased at a rate of 3˚C/min to 230˚C which will be maintained for 5 min. The total run time will be 33 min. The FID hydrogen flow was 30 ml/min and air flow 400 ml /min. Hydrogen and air will be used for flame ionization detection. The carrier and make-up gas will be hydrogen at a flow rate of 1.5 ml/min and 25 ml/min, respectively. The aliquots of 12 hr and 24 hour urine samples will be thawed and mixed using a vortex. Two test tubes each containing of 200 μl, of the urine sample will be prepared in parallel. Forthy μl of internal standard containing 20 mg/ml of Phenyl Beta-D-Glucoside, and 20 mg/ml of myo-Inositol will be added to both tubes while a known amount of four sugar probe will be added to only one of the tubes. The mixture will be evaporated to dryness at 70˚C under a stream of

nitrogen. The dried residues will be taken up in 200 μl of anhydrous pyridine containing 25 mg/ml of hydroxylamine, mixed, heated at 70°C for 1 h, and centrifuged at 2250 rpm for 5 min. An aliquot (100 μl) of the supernatant will be transferred to a small conical tube and the sugar oximes will be silylated with 100 μl of N-trimethylsilylimidazole for 30 min at 70°C. An aliquot (100 μl) of the silylated derivatives will be sealed in an autosampler vial for testing. We will be calculating sugar concentrations in patients' urine samples based on slope and intercept of the line created by each pair of spiked and non-spiked urine samples. We will use this method to decrease the variability we observed in different urine samples when they were spiked with the same amount of sugar. In this method, the sugar concentration in each sample is expected to be equal to intercept/coefficient ratio. The total amounts of each sugar in the 12-h and 24-h urine samples will be expressed as percentages of the amounts of sugar that were ingested in the oral dose. The respective data will be analyzed using SPSS. All values for 12 hour and 24 hour urine excretions will be standardized using control subjects ([Sugar level-μ]/SD).

2. LH- PCR Fingerprint Analysis: Total genomic DNA will be extracted from samples using Bio101 kit from Qbiogene, Inc., Montreal, Quebec. Extracted DNA (10 ng) is amplified by PCR using a fluorescently labeled forward primer 27F (5'-[6FAM] AGAGTTTGATCCTGGCTCA G-3') and unlabeled reverse primer 355R' (5'-GCTGCCTCCCGTAGGAGT-3'). Both primers are universal primers for Bacteria. The reactions are performed using 20-ul final volume containing 1 X PCR buffer, 1.5 mM MgCl2, 0.2 mM of each deoxynucleoside triphosphate, 0.2 uM of each primer, and 2 U of Taq DNA polymerase. The initial denaturation step was 95°C for 11 minutes, followed by 35 cycles consisting of denaturation at 95°C for 30 seconds, annealing at 48°C for 30 seconds, extension at 72°C for 2 minutes, with a final extension step that consists of 72°C for 20 minutes. The LH-PCR amplification efficiency will be quantified by agarose gel electrophoresis.

The LH-PCR products will be diluted according to their intensity on the agarose gel and mixed with ILS-600 size standards (ABI) and HiDi Formamide (ABI). The diluted samples will be separated on the SCE9610 capillary fluorescent sequencer (Spectrumedix LLC, State College, PA) and analyzed with the GenoSpectrum™ software package (Version 2.01) which deconvolves the fluorescence data into electropherograms where the peaks of the electropherograms represent PCR amplicons of different length in base pairs (bp) which represent different species or Operational Taxanomic Units (OTU) of microflora. All LH-PCR fingerprinting data will be analyzed using a custom PERL script (Interleave 1.0, BioSpherex LLC) that combines data from several runs,

interleaves the various profiles, normalizes the data, calculates the averages for each amplicon size for the disease state (i.e. averages for IBD and controls samples), and determines diversity indices. The normalized peak areas (abundance proportions) will be calculated by dividing an individual peak area by the total peak area in that profile. A subset of the samples with the highest levels of diversity will then selected for future pyrosequencing based on this latter analysis, as needed for future analysis. Part of the microbiota analysis will be performed at George Mason University, through the research laboratories of Prof. Patrick Gillevet, Ph.D. All samples sent to his labs will be stripped of any patient identifier information and will be marked with only a created subject number for this study.

3. Histopathology and Immunohistochemistry Analysis: a portion of the colon tissue collected as part of the study will be utilized and processed with α-synuclein staining to detect alpha-synuclein pathology and inflammation and to measure cytokines and neurotoxin proteins. The process will be done by Dr. Jeffrey H. Kordower in his lab at Rush University Medical Center.

Risks and benefits:

This study will not directly benefit study participants. There is a chance that information learned from this study will help to explain the causes of Parkinson's disease or multiple system, atrophy.

There is a minor risk of bleeding and perforation with any endoscopic procedure, including flexible sigmoidoscopy. These risks in the case of clinical sigmoidoscopy that is inserted to 60 cm from the anal verge occur as a result of bowel prep (1/1000 or less), from bleeding from interventions (less than 1%) and perforation is extremely rare at 1/25000 to 1/50000. Typical bleeding occurs after polyp removal especially after cautery using snare or a hot biopsy forceps. Clinically up to 50 biopsies can be obtained and the number of biopsies does not pose a risk to the subjects.

There may be slight rectal discomfort with rectal swabs comparable to obtaining a rectal temperature.

The urine collection may be cumbersome or embarrassing.

Procedures to reduce risks:

To minimize these risks, subjects will NOT be given any bowel preps, the sigmoidoscope will only be inserted ONLY to 20 cm until biopsies could be safely obtained, no cautery will be employed for biopsies, and every effort will be taken to be very careful with the cold biopsy forceps. The procedures will only be performed by Drs. Keshavarzian and Mutlu who have performed these type of procedures extensively for clinical and research purposes. The risk of perforation is related to the depth of insertion of the scope and the highest due to looping of the scope by a significant insertion depth. Therefore, limiting the depth of insertion to 20 cm will minimize this potential risk significantly close to comparable to taking an enema. This method represents the safest possible method to obtain mucosal samples from subjects.

Subjects will be telephoned one week after their sigmoidoscopy by the GI research assistants to assure no adverse events have happened. All serious adverse events will be reported to the GI endoscopy quality assurance committee and to Dr. John Losurdo, M.D. (Director of the GI endoscopy labs) as well as the IRB as soon as possible within 24 hours.

<u>Subject compensation and Subject benefits:</u>

Subjects will be compensated a total of 150 dollars for their time and effort for this study. They will receive the first 75 dollars after completion of the sigmoidoscopy (visit 2 or 3) and the 2nd 75 dollars after completion of their 1 month visit (last visit). Subjects will not be charged for any of their visits and will be given a parking sticker.

The limited unprepped flexible sigmoidoscopy performed for research purposes for this study will not be adequate to be acceptable for colon cancer screening. However, there may be some direct benefits of a limited flexible sigmoidoscopy to both the study and control groups. For example identification of significant lesions (albeit low chance in those under the age of 50) at this limited sigmoidoscopy exam, may prompt a colonoscopy that may result in early diagnosis and treatment of colonic pathology. If healthy volunteers are recruited as needed, they will receive a complete GI physician evaluation with a complete history and physical exam and blood tests (CBC and CMP) to verify that they are healthy.

REFERENCES

1. Abbott RD, Petrovitch H, White LR, Masaki KH, Tanner CM, Curb JD, et al. Frequency of bowel movements and the future risk of Parkinson's disease. Neurology 2001;57(3):456-462.
2. Abbott RD, Ross GW, White LR, Sanderson WT, Burchfiel CM, Kashon M, et al. Environmental, life-style, and physical precursors of clinical Parkinson's disease: recent findings from the Honolulu-Asia Aging Study. J Neurol 2003;250 Suppl 3:III30-39.
3. Abbott RD, Ross GW, White LR, Tanner CM, Masaki KH, Nelson JS, et al. Excessive daytime sleepiness and subsequent development of Parkinson disease. Neurology 2005;65(9):1442-1446.
4. Barbic F, Perego F, Canesi M, Gianni M, Biagiotti S, Costantino G, et al. Early abnormalities of vascular and cardiac autonomic control in Parkinson's disease without orthostatic hypotension. Hypertension 2007;49(1):120-126.
5. Braak H, Rub U, Gai WP, Del Tredici K. Idiopathic Parkinson's disease: possible routes by which vulnerable neuronal types may be subject to neuroinvasion by an unknown pathogen. J Neural Transm 2003;110(5):517-536.
6. Hardy J, Cai H, Cookson MR, Gwinn-Hardy K, Singleton A. Genetics of Parkinson's disease and parkinsonism. Ann Neurol 2006;60(4):389-398.
7. Liu B, Gao HM, Hong JS. Parkinson's disease and exposure to infectious agents and pesticides and the occurrence of brain injuries: role of neuroinflammation. Environ Health Perspect 2003;111(8):1065-1073.
8. Backhed F, Ley RE, Sonnenburg JL, Peterson DA, Gordon JI. Host-bacterial mutualism in the human intestine. Science 2005;307(5717):1915-1920.
9. Seksik P, Rigottier-Gois L, Gramet G, Sutren M, Pochart P, Marteau P, et al. Alterations of the dominant faecal bacterial groups in patients with Crohn's disease of the colon. Gut 2003;52(2):237-242.
10. Palmer C, Bik EM, Digiulio DB, Relman DA, Brown PO. Development of the Human Infant Intestinal Microbiota. PLoS Biol 2007;5(7):e177.
11. Davies KN, King D, Billington D, Barrett JA. Intestinal permeability and orocaecal transit time in elderly patients with Parkinson's disease. Postgrad Med J 1996;72(845):164-167.

Appendices

Unified Parkinson's Disease Rating Scale
Hoehn & Yahr Stage
MMSE
GI Symptom Checklist for Healthy Control Subjects
GI Symptom Checklist for Parkinson's Disease Subjects

Unified Parkinson Disease Rating Scale (UPDRS)

I. **Mentation, Behavior, Mood**
  - **Intellectual Impairment**

0-none
1-mild (consistent forgetfulness with partial recollection of events with no other difficulties)
2-moderate memory loss with disorientation and moderate difficulty handling complex problems
3-severe memory loss with disorientation to time and often place, severe impairment with problems
4-severe memory loss with orientation only to person, unable to make judgments or solve problems

  - **Thought Disorder**

0-none
1-vivid dreaming
2-"benign" hallucination with insight retained
3-occasional to frequent hallucination or delusions without insight, could interfere with daily activities
4-persistent hallucination, delusions, or florid psychosis.

  - **Depression**

0-not present
1-periods of sadness or guilt greater than normal, never sustained for more than a few days or a week
2-sustained depression for >1 week
3-vegetative symptoms (insomnia, anorexia, abulia, weight loss)
4-vegetative symptoms with suicidality

  - **Motivation/Initiative**

0-normal
1-less of assertive, more passive
2-loss of initiative or disinterest in elective activities
3-loss of initiative or disinterest in day to say (routine) activities
4-withdrawn, complete loss of motivation

II. **Activities of Daily Living**
  - **Speech**

0-normal

1-mildly affected, no difficulty being understood
2-moderately affected, may be asked to repeat
3-severely affected, frequently asked to repeat
4-unintelligible most of time

- **Salivation**

0-normal
1-slight but noticeable increase, may have nighttime drooling
2-moderately excessive saliva, hay minimal drooling
3-marked drooling

- **Swallowing**

0-normal
1-rare choking
2-occasional choking
3-requires soft food
4-requires NG tube or G-tube

- **Handwriting**

0-normal
1-slightly small or slow
2-all words small but legible
3-severely affected, not all words legible
4-majority illegible

- **Cutting Food/Handing Utensils**

0-normal
1-somewhat slow and clumsy but no help needed
2-can cut most foods, some help needed
3-food must be cut, but can feed self
4-needs to be fed

- **Dressing**

0-normal
1-somewhat slow, no help needed
2-occasional help with buttons or arms in sleeves
3-considerable help required but can do something alone
4-helpless

- **Hygiene**

0-normal
1-somewhat slow but no help needed
2-needs help with shower or bath or very slow in hygienic care
3-requires assistance for washing, brushing teeth, going to bathroom
4-helpless

- **Turning in Bed/ Adjusting Bed Clothes**

0-normal
1-somewhat slow no help needed
2-can turn alone or adjust sheets but with great difficulty
3-san initiate but not turn or adjust alone
4-helpless

- **Falling-Unrelated to Freezing**

0-none
1-rare falls
2-occasiona, less than one per day
3-average of once per day
4->1 per day

- **Freezing When Walking**

0-normal
1-rare, may have start hesitation
2-occasional falls from freezing,
3-frequent freezing, occasional falls
4-frequent falls from freezin

- **Walking**

0-normal
1-mild difficulty, day drag legs or decrease arm swing
2-moderate difficultly requires no assist
3-severe disturbance requires assistance
4-cannot walk at all even with assist

- **Tremor**

0-absent
1-slight and infrequent, not bothersome to patient
2-moderate, bothersome to patient
3-severe, interfere with many activities
4-marked, interferes with many activities

- **Sensory Complaints Related to Parkinsonism**

0-none
1-occasionally has numbness, tingling, and mild aching
2-frequent, but not distressing
3-frequent painful sensation
4-excruciating pain

III. **Motor Exam**

- **Speech**

0-normal
1-slight loss of expression, diction,volume
2-monotone, slurred but understandable, mod. impaired

3-marked impairment, difficult to understand
4-unintelligible

- **Facial Expression**

0-Normal
1-slight hypomymia, could be poker face
2-slight but definite abnormal diminution in expression
3-mod. hypomimia, lips parted some of time
4-masked or fixed face, lips parted 1/4 of inch or more with complete loss of expression

- **Tremor at Rest**
  - **Face**

0-absent
1-slight and infrequent
2-mild and present most of time
3-moderate and present most of time
4-marked and present most of time

  - **Right Upper Extremity (RUE)**

0-absent
1-slight and infrequent
2-mild and present most of time
3-moderate and present most of time
4-marked and present most of time

  - **LUE**

0-absent
1-slight and infrequent
2-mild and present most of time
3-moderate and present most of time
4-marked and present most of time

  - **RLE**

0-absent
1-slight and infrequent
2-mild and present most of time
3-moderate and present most of time
4-marked and present most of time

  - **LLE**

0-absent
1-slight and infrequent
2-mild and present most of time
3-moderate and present most of time
4-marked and present most of time

- **Action or Postural Tremor**

- **RUE**

0-absent
1-slight, present with action
2-moderate, present with action
3-moderate present with action and posture holding
4-marked, interferes with feeding

- **LUE**

0-absent
1-slight, present with action
2-moderate, present with action
3-moderate present with action and posture holding
4-marked, interferes with feeding

- **Rigidity**
  - **Neck**

0-absent
1-slight or only with activation
2-mild/moderate
3-marked, full range of motion
4-severe

- **RUE**

0-absent
1-slight or only with activation
2-mild/moderate
3-marked, full range of motion
4-severe

- **LUE**

0-absent
1-slight or only with activation
2-mild/moderate
3-marked, full range of motion
4-severe

- **RLE**

0-absent
1-slight or only with activation
2-mild/moderate
3-marked, full range of motion
4-severe

- **LLE**

0-absent

1-slight or only with activation
2-mild/moderate
3-marked, full range of motion
4-severe

- **Finger taps**
  - **Right**

0-normal
1-mild slowing, and/or reduction in amp.
2-moderate impaired. Definite and early fatiguing, may have occasional arrests
3-severely impaired. Frequent hesitations and arrests.
4-can barely perform

  - **Left**

0-normal
1-mild slowing, and/or reduction in amp.
2-moderate impaired. Definite and early fatiguing, may have occasional arrests
3-severely impaired. Frequent hesitations and arrests.
4-can barely perform

- **Hand Movements (open and close hands in rapid succession)**
  - **Right**

0-normal
1-mild slowing, and/or reduction in amp.
2-moderate impaired. Definite and early fatiguing, may have occasional arrests
3-severely impaired. Frequent hesitations and arrests.
4-can barely perform

  - **Left**

0-normal
1-mild slowing, and/or reduction in amp.
2-moderate impaired. Definite and early fatiguing, may have occasional arrests
3-severely impaired. Frequent hesitations and arrests.
4-can barely perform

- **Rapid Alternating Movements (pronate and supinate hands)**
  - **Right**

0-normal
1-mild slowing, and/or reduction in amp.
2-moderate impaired. Definite and early fatiguing, may have occasional arrests
3-severely impaired. Frequent hesitations and arrests.
4-can barely perform

  - **Left**

0-normal
1-mild slowing, and/or reduction in amp.

2-moderate impaired. Definite and early fatiguing, may have occasional arrests
3-severely impaired. Frequent hesitations and arrests.
4-can barely perform

- **Leg Agility (tap heel on ground, amp should be 3 inches)**
  - **Right**

0-normal
1-mild slowing, and/or reduction in amp.
2-moderate impaired. Definite and early fatiguing, may have occasional arrests
3-severely impaired. Frequent hesitations and arrests.
4-can barely perform

  - **Left**

0-normal
1-mild slowing, and/or reduction in amp.
2-moderate impaired. Definite and early fatiguing, may have occasional arrests
3-severely impaired. Frequent hesitations and arrests.
4-can barely perform

- **Arising From Chair (pt. arises with arms folded across chest)**

0-normal
1-slow, may need more than one attempt
2-pushes self up from arms or seat
3-tends to fall back, may need multiple tries but can arise without assistance
4-unable to arise without help

- **Posture**

0-normal erect
1-slightly stooped, could be normal for older person
2-definitely abnormal, mod. stooped, may lean to one side
3-severely stooped with kyphosis
4-marked flexion with extreme abnormality of posture

- **Gait**

0-normal
1-walks slowly, may shuffle with short steps, no festination or propulsion
2-walks with difficulty, little or no assistance, some festination, short steps or propulsion
3-severe disturbance, frequent assistance
4-cannot walk

- **Postural Stability (retropulsion test)**

0-normal
1-recovers unaided
2-would fall if not caught
3-falls spontaneously
4-unable to stand

- **Body Bradykinesia/ Hypokinesia**

0-none
1-minimal slowness, could be normal, deliberate character
2-mild slowness and poverty of movement, definitely abnormal, or dec. amp. of movement
3-moderate slowness, poverty, or small amplitude
4-marked slowness, poverty, or amplitude

GI SYMPTOM AND SEVERITY CHECKLIST
FOR LIPOPOLYSACCHARIDE PROJECT

1. On the number scale, please circle the number that corresponds to the severity of each symptom (1 being very mild and 10 being very severe). If the symptom in not present, please circle 0:

example: sneezing

Not present ... *Very severe*
0 1 2 3 4 5 6 7 8 9 10

How long have you had this?

Upper abdominal pain or discomfort

Not present *Very mild* ... *Very mild*
0 1 2 3 4 5 6 7 8 9 10

_____Years _____ Months

Lower abdominal pain or discomfort

Not present *Very mild* ... *Very severe*
0 1 2 3 4 5 6 7 8 9 10

_____Years _____ Months

Upper abdominal cramping

Not present *Very mild* ... *Very severe*
0 1 2 3 4 5 6 7 8 9 10

_____Years _____ Months

Lower abdominal cramping

Not present *Very mild* ... *Very severe*
0 1 2 3 4 5 6 7 8 9 10

_____Years _____ Months

Bloating

Not present *Very mild* ... *Very severe*
0 1 2 3 4 5 6 7 8 9 10

_____Years _____ Months

Excessive Belching

Not present *Very mild* ... *Very severe*
0 1 2 3 4 5 6 7 8 9 10

_____Years _____ Months

Excessive gas passage (flatulence)

Not present *Very mild* ... *Very severe*
0 1 2 3 4 5 6 7 8 9 10

_____Years _____ Months

Excessive gas overall

Not present *Very mild* ... *Very severe*
0 1 2 3 4 5 6 7 8 9 10

_____Years _____ Months

Heartburn

Not present *Very mild* ... *Very severe*
0 1 2 3 4 5 6 7 8 9 10

_____Years _____ Months

Indigestion

Not present *Very mild* ... *Very severe*
0 1 2 3 4 5 6 7 8 9 10

_____Years _____ Months

Nausea

Not present *Very mild* ... *Very severe*
0 1 2 3 4 5 6 7 8 9 10

_____Years _____ Months

Vomiting

Not present *Very mild* ... *Very severe*
0 1 2 3 4 5 6 7 8 9 10

_____Years _____ Months

Alternating bowel movements (alternating between constipation and diarrhea)

_____Years _____ Months

Revised: 3/28/2011

Not present — *Very mild* — *Very severe*
0 1 2 3 4 5 6 7 8 9 10

Constipation majority of the time _____Years _____ Months

Not present — *Very mild* — *Very severe*
0 1 2 3 4 5 6 7 8 9 10

Diarrhea majority of the time _____Years _____ Months

Not present — *Very mild* — *Very severe*
0 1 2 3 4 5 6 7 8 9 10

Irregular bowel habits _____Years _____ Months

Not present — *Very mild* — *Very severe*
0 1 2 3 4 5 6 7 8 9 10

Infrequent bowel movements _____Years _____ Months

Not present — *Very mild* — *Very severe*
0 1 2 3 4 5 6 7 8 9 10

Very frequent bowel movements _____Years _____ Months

Not present — *Very mild* — *Very severe*
0 1 2 3 4 5 6 7 8 9 10

Hard stools _____Years _____ Months

Not present — *Very mild* — *Very severe*
0 1 2 3 4 5 6 7 8 9 10

Watery or very soft/loose stools _____Years _____ Months

Not present — *Very mild* — *Very severe*
0 1 2 3 4 5 6 7 8 9 10

Passage of mucous in the stool _____Years _____ Months

Not present — *Very mild* — *Very severe*
0 1 2 3 4 5 6 7 8 9 10

Passage of blood in the stool (without the presence of hemorrhoids) _____Years _____ Months

Not present — *Very mild* — *Very severe*
0 1 2 3 4 5 6 7 8 9 10

Straining with bowel movements _____Years _____ Months

Not present — *Very mild* — *Very severe*
0 1 2 3 4 5 6 7 8 9 10

Urgency to have a bowel movement _____Years _____ Months

Not present — *Very mild* — *Very severe*
0 1 2 3 4 5 6 7 8 9 10

Bowel incontinence (loss of control of stool) _____Years _____ Months

Not present — *Very mild* — *Very severe*
0 1 2 3 4 5 6 7 8 9 10

Sensation of incomplete emptying of bowels at end of bowel movement _____Years _____ Months

Not present — *Very mild* — *Very severe*
0 1 2 3 4 5 6 7 8 9 10

Loss of appetite _____Years _____ Months

Not present — *Very mild* — *Very severe*
0 1 2 3 4 5 6 7 8 9 10

Weight loss _____Years _____ Months

Not present *Very mild* *Very severe*
0 1 2 3 4 5 6 7 8 9 10

Decreased food intake because of digestive symptoms ______Years ______ Months

Not present *Very mild* *Very severe*
0 1 2 3 4 5 6 7 8 9 10

Difficulty swallowing ______Years ______ Months

Not present *Very mild* *Very severe*
0 1 2 3 4 5 6 7 8 9 10

Pain with swallowing ______Years ______ Months

Not present *Very mild* *Very severe*
0 1 2 3 4 5 6 7 8 9 10

Food coming up to mouth ______Years ______ Months

Not present *Very mild* *Very severe*
0 1 2 3 4 5 6 7 8 9 10

Acid taste in mouth ______Years ______ Months

Not present *Very mild* *Very severe*
0 1 2 3 4 5 6 7 8 9 10

Intolerance/allergy to multiple foods ______Years ______ Months

Not present *Very mild*
0 1 2 3 4 5 6 7 8 9 10

2. How often do you have bowel movements? (example: once a day, twice a week, etc.)

____

3. Most of the time your bowel movements look like: (please circle Type)

Type 1	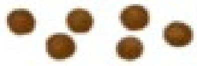	Separate hard lumps, like nuts (hard to pass)
Type 2	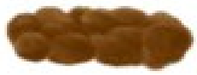	Sausage-shaped but lumpy
Type 3	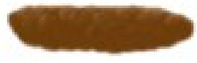	Like a sausage but with cracks on its surface
Type 4	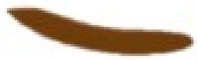	Like a sausage or snake, smooth and soft
Type 5	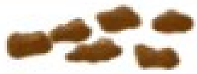	Soft blobs with clear-cut edges (passed easily)
Type 6	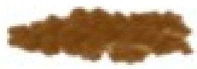	Fluffy pieces with ragged edges, a mushy stool
Type 7	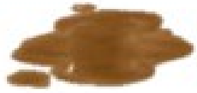	Watery, no solid pieces. **Entirely Liquid**

GI SYMPTOM AND SEVERITY CHECKLIST FOR LIPOPOLYSACCHARIDE

4. On the number scale, please circle the number that corresponds to the severity of each symptom (1 being very mild and 10 being very severe). If the symptom in not present, please circle 0.

example: sneezing

Not present 0 — *Very sever* 1 — 2 — 3 — 4 — 5 — 6 — 7 — 8 — 9 — 10 *Very severe*

	How long have you had this?	This problem began before or after Parkinson's began? (Circle one)
Upper abdominal pain or discomfort<br>*Not present* 0 1 *Very mild* 2 3 4 5 6 7 8 9 10	_____Years _____ Months	Before After
Lower abdominal pain or discomfort<br>*Not present* 0 1 *Very mild* 2 3 4 5 6 7 8 9 10 *Very severe*	_____Years _____ Months	Before After
Upper abdominal cramping<br>*Not present* 0 1 *Very mild* 2 3 4 5 6 7 8 9 10 *Very severe*	_____Years _____ Months	Before After
Lower abdominal cramping<br>*Not present* 0 1 *Very mild* 2 3 4 5 6 7 8 9 10 *Very severe*	_____Years _____ Months	Before After
Bloating<br>*Not present* 0 1 *Very mild* 2 3 4 5 6 7 8 9 10 *Very severe*	_____Years _____ Months	Before After
Excessive Belching<br>*Not present* 0 1 *Very mild* 2 3 4 5 6 7 8 9 10 *Very severe*	_____Years _____ Months	Before After
Excessive gas passage (flatulence)<br>*Not present* 0 1 *Very mild* 2 3 4 5 6 7 8 9 10 *Very severe*	_____Years _____ Months	Before After
Excessive gas overall<br>*Not present* 0 1 *Very mild* 2 3 4 5 6 7 8 9 10 *Very severe*	_____Years _____ Months	Before After
Heartburn<br>*Not present* 0 1 *Very mild* 2 3 4 5 6 7 8 9 10 *Very severe*	_____Years _____ Months	Before After
Indigestion<br>*Not present* 0 1 *Very mild* 2 3 4 5 6 7 8 9 10 *Very severe*	_____Years _____ Months	Before After
Nausea<br>*Not present* 0 1 *Very mild* 2 3 4 5 6 7 8 9 10 *Very severe*	_____Years _____ Months	Before After
Vomiting<br>*Not present* 0 1 *Very mild* 2 3 4 5 6 7 8 9 10 *Very severe*	_____Years _____ Months	Before After

Alternating bowel movements (alternating between constipation and diarrhea)

Not present *Very mild* *Very severe*
0 1 2 3 4 5 6 7 8 9 10

_____Years _____ Months

Before After

Constipation majority of the time

Not present *Very mild* *Very severe*
0 1 2 3 4 5 6 7 8 9 10

_____Years _____ Months

Before After

Diarrhea majority of the time

Not present *Very mild* *Very severe*
0 1 2 3 4 5 6 7 8 9 10

_____Years _____ Months

Before After

Irregular bowel habits

Not present *Very mild* *Very severe*
0 1 2 3 4 5 6 7 8 9 10

_____Years _____ Months

Before After

Infrequent bowel movements

Not present *Very mild* *Very severe*
0 1 2 3 4 5 6 7 8 9 10

_____Years _____ Months

Before After

Very frequent bowel movements

Not present *Very mild* *Very severe*
0 1 2 3 4 5 6 7 8 9 10

_____Years _____ Months

Before After

Hard stools

Not present *Very mild* *Very severe*
0 1 2 3 4 5 6 7 8 9 10

_____Years _____ Months

Before After

Watery or very soft/loose stools

Not present *Very mild* *Very severe*
0 1 2 3 4 5 6 7 8 9 10

_____Years _____ Months

Before After

Passage of mucous in the stool

Not present *Very mild* *Very severe*
0 1 2 3 4 5 6 7 8 9 10

_____Years _____ Months

Before After

Passage of blood in the stool (without the presence of hemorrhoids)

Not present *Very mild* *Very severe*
0 1 2 3 4 5 6 7 8 9 10

_____Years _____ Months

Before After

Straining with bowel movements

Not present *Very mild* *Very severe*
0 1 2 3 4 5 6 7 8 9 10

_____Years _____ Months

Before After

Urgency to have a bowel movement

Not present *Very mild* *Very severe*
0 1 2 3 4 5 6 7 8 9 10

_____Years _____ Months

Before After

Bowel incontinence (loss of control of stool)

Not present *Very mild* *Very severe*
0 1 2 3 4 5 6 7 8 9 10

_____Years _____ Months

Before After

Sensation of incomplete emptying of bowels at end of bower movement

Not present *Very mild* *Very severe*
0 1 2 3 4 5 6 7 8 9 10

_____Years _____ Months

Before After

Loss of appetite

Not present *Very mild* *Very severe*
0 1 2 3 4 5 6 7 8 9 10

_____Years _____ Months

Before After

Weight loss

Not present *Very mild* *Very severe*
0 1 2 3 4 5 6 7 8 9 10

_____Years _____ Months

Before After

Decreased food intake because of digestive symptoms

Not present *Very mild* *Very severe*
0 1 2 3 4 5 6 7 8 9 10

_____Years _____ Months

Before After

Difficulty swallowing

Not present *Very mild* *Very severe*
0 1 2 3 4 5 6 7 8 9 10

_____Years _____ Months

Before After

Pain with swallowing

Not present *Very mild* *Very severe*
0 1 2 3 4 5 6 7 8 9 10

_____Years _____ Months

Before After

Food coming up to mouth

Not present *Very mild* *Very severe*
0 1 2 3 4 5 6 7 8 9 10

_____Years _____ Months

Before After

Acid taste in mouth

Not present *Very mild* *Very severe*
0 1 2 3 4 5 6 7 8 9 10

_____Years _____ Months

Before After

Intolerance/allergy to multiple foods

Not present *Very mild* *Very severe*
0 1 2 3 4 5 6 7 8 9 10

_____Years _____ Months

Before After

5. How often do you have bowel movements? (example: once a day, twice a week, etc.)

____

6. Most of the time your bowel movement look like: (please circle Type)

Type 1	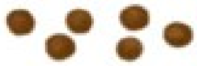	Separate hard lumps, like nuts (hard to pass)
Type 2	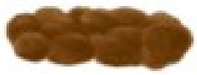	Sausage-shaped but lumpy
Type 3	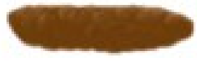	Like a sausage but with cracks on its surface
Type 4	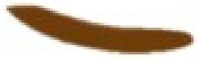	Like a sausage or snake, smooth and soft
Type 5	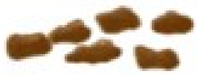	Soft blobs with clear-cut edges (passed easily)
Type 6	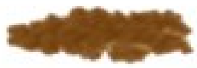	Fluffy pieces with ragged edges, a mushy stool
Type 7	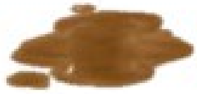	Watery, no solid pieces. **Entirely Liquid**
